# Therapeutic potential of CKD-506, a novel selective histone deacetylase 6 inhibitor, in a murine model of rheumatoid arthritis

**DOI:** 10.1186/s13075-020-02258-0

**Published:** 2020-07-25

**Authors:** Jin Kyun Park, Yu Jin Jang, Bo Ram Oh, Jieun Shin, Daekwon Bae, Nina Ha, Young il Choi, Gi Soo Youn, Jinseu Park, Eun Young Lee, Eun Bong Lee, Yeong Wook Song

**Affiliations:** 1 Division of Rheumatology, Department of Internal Medicine, Seoul National University College of Medicine, Seoul, South Korea; 2 Department of Molecular Medicine and Biopharmaceutical Sciences, Graduate School of Convergence Science and Technology, Seoul National University, Seoul, South Korea; 3 Department of Pharmacology and Toxicology, CKD Research Institute, CKD Pharmaceutical Company, Seoul, South Korea; 4 Department of Biomedical Science and Research Institute for Bioscience & Biotechnology, Hallym University, Chuncheon, South Korea

**Keywords:** Rheumatoid arthritis, HDAC6, Inflammation, Inhibitor, Drug

## Abstract

**Objectives:**

Histone deacetylase (HDAC) 6 promotes inflammation. We investigated the anti-arthritic effects of CKD-506, a novel HDAC6 inhibitor, in vitro and in a murine model of arthritis as a novel treatment option for rheumatoid arthritis (RA).

**Methods:**

HDAC6 was overexpressed in mouse peritoneal macrophages and RAW 264.7 cells, and the effects of a HDAC6 inhibitor CKD-506 on cytokine production and activity of NF-κB and AP-1 signaling were examined. Peripheral blood mononuclear cells (PBMCs) from RA patients and fibroblast-like synoviocytes (FLS) were activated in the presence of CKD-506. Next, regulatory T cells (Tregs) were induced from RA patients and co-cultured with healthy effector T cells (Teffs) and cell proliferation was analyzed by flow cytometry. Finally, the effects of the inhibitor on the severity of arthritis were assessed in a murine model of adjuvant-induced arthritis (AIA).

**Results:**

Overexpression of HDAC6 induced macrophages to produce TNF-α and IL-6. The inhibitory effect of CKD-506 was mediated via blockade of NF-κB and AP-1 activation. HDAC6 inhibition reduced TNF-α and IL-6 production by activated RA PBMCs. CKD-506 inhibited production of MMP-1, MMP-3, IL-6, and IL-8 by activated FLS. In addition, CKD-506 inhibited proliferation of Teffs directly and indirectly by improving iTreg function. In AIA rats, oral CKD-506 improved clinical arthritis in a dose-dependent manner. A combination of sub-therapeutic CKD-506 and methotrexate exerted a synergistic effect.

**Conclusion:**

The novel HDAC6 inhibitor CKD-506 suppresses inflammatory responses by monocytes/macrophages, improves Treg function, and ameliorates arthritis severity in a murine model of RA. Thus, CKD-506 might be a novel and effective treatment option for RA.

## Introduction

Rheumatoid arthritis (RA) is an autoimmune disease characterized by chronic inflammation and destruction of joints [1]. Activated immune cells produce inflammatory cytokines, including tumor necrosis factor (TNF-α), interleukin (IL)-1β, and IL-6, which drive inflammatory responses and induce fibroblast-like synoviocytes (FLS) to produce tissue-destructive matrix metalloproteinases (MMPs) and chemokines. These inflammatory mediators, in turn, recruit immune cells and potentiate joint destruction [2, 3]. Regulatory T (Treg) cells, which counteract “overshooting” of the immune response, are compromised in RA patients [4]. Therefore, a treatment that targets multiple key steps in RA pathogenesis might be more effective than those that target a single pathway. A potential treatment is inhibition of histone deacetylase (HDAC) [5, 6].

While nuclear HDAC is critical for epigenetic regulation, which ultimately determines cell differentiation and function, cytosolic HDACs are involved in post-translational modification of non-histone proteins in the cytosol, which are essential for cellular functions such as intra-cellular transport and cell migration among others [7]. HDAC activity in RA patients is higher than that in healthy controls, and HDAC inhibitors are effective in murine models of RA [8–12]. Non-specific pan-HDAC inhibitors, which suppress multiple isoforms of HDAC with pleiotropic effects, have anti-inflammatory properties; however, they are also associated with side effects such as fatigue, diarrhea, nausea, and neutropenia [13, 14]. Therefore, selective inhibition of a specific HDAC isoform might offer substantial advantages with a better safety margin. HDAC6 is different from other HDAC isotypes in that it is present almost exclusively in the cytosol and, therefore, is not involved in epigenetic regulation [15, 16]. Overexpression of HDAC6 is associated with increased inflammatory responses [17], and inhibiting it reduces disease activity in murine model of RA and systemic lupus erythematosus [18, 19]. CKD-506 is a potent and selective HDAC6 inhibitor (> 100-fold selectivity for HDAC6 over other HDAC isotypes). It induced tubulin acetylation in a dose-dependent manner without affecting histone H4 acetylation in both human and murine cells [20]. CKD-506 ameliorated acute and chronic murine colitis and lupus nephritis in animal models [20, 21].

The aim of this study was to investigate the therapeutic effects of CKD-506, a novel HDAC6 inhibitor, as a potential drug candidate for the treatment of RA. We show that inhibiting HDAC6 with CKD-506 improves Treg function, suppresses inflammatory responses by macrophages and FLS, and attenuates arthritis in a murine model of adjuvant-induced arthritis (AIA).

## Materials and methods

### Cell preparation

Resident peritoneal macrophages were isolated from 6-week-old male ICR mice (Experimental Animal Center, Hallym University) as described previously [17]. Peripheral blood mononuclear cells (PBMCs) were isolated from the blood of RA patients by Ficoll-Hypaque density centrifugation and resuspended in RPMI-1640 containing 1% fetal bovine serum and 1% penicillin/streptomycin. FLS, isolated from synovial tissue taken from RA patients, were cultured in DMEM containing 10% fetal bovine serum and 1% penicillin/streptomycin. For all experiments, cells were used between passages 3 and 7. The viability of PBMCs and FLS was evaluated after incubation for 2 h with CCK-8 (DOJINDO, Kumamoto, Japan). Optical density was read at 450 nm.

### Transient transfection and luciferase assay

RAW 264.7 cells were transfected with a pcDNA3.1 control vector or a HDAC6 expression vector (pcDNA-HDAC6-FLAG) using Lipofectamine 3000 reagent (Thermo Fisher Scientific). For the reporter assays, cells were co-transfected with a pNF-κB-luc or pAP-1-luc plasmid (Stratagene, La Jolla, CA, USA) and a control (pCMV-β-galactosidase) plasmid using Lipofectamine 3000 reagent as previously described [17]. Cell lysates were prepared, and luciferase and β-galactosidase activity analyzed. The luciferase activity of each sample was normalized to that of β-galactosidase, and the results were expressed as a fold change in transactivation.

#### Measurement of cytokines in cell culture supernatants

RA PBMCs were treated with HDAC6 inhibitors and then stimulated with LPS. RA FLS were treated with HDAC6 inhibitors and then stimulated with IL-1β. After 24 h, cell culture supernatants were collected and the amounts of TNF-α, IL-1β, IL-6, and IL-10 secreted by PBMCs and the amounts of MMP-1, MMP-3, IL-6, and IL-8 secreted by FLS were measured in enzyme-linked immunosorbent assays (ELISAs).

#### Cell proliferation assay

CD4+ CD25− T cells were purified from healthy PBMCs by negative selection using a CD4+ T Cell Biotin-Antibody Cocktail (Miltenyi Biotec). Induced Treg (iTreg) cells were generated from CD4+ CD25− T cells of RA patients in the presence of an anti-CD3 antibody (eBioscience, San Diego, CA, USA), an anti-CD28 antibody (BD Pharmingen, San Diego, CA, USA), IL-2 (PEPROTECH), TGF-β (PEPROTECH), and vitamin D3 (SIGMA) [15]. CD4+ CD25− T cells from healthy controls were labeled for 10 min with 5 mM carboxyfluorescein diacetate succinimidyl ester (CFSE) (Life Technologies, Eugene, OR, USA). RA iTreg cells and CFSE-labeled healthy effector T cells were co-cultured for 72 h at a ratio of 0:1, 0.3:1, and 1:1 in the presence of Dynabeads Human T-Activator CD3/CD28 (Invitrogen Dynal AS, Life Technologies, Oslo, Norway). T cell proliferation was measured by flow cytometry.

### Induction of experimental arthritis in rats

All animal experiments were approved by the Animal Care and Use Committee. Lewis rats (female, 5 weeks old) were purchased from Central Lab Animal, Inc. (Seoul, Korea). Complete Freund’s adjuvant (CFA) (Chondrex, Seattle, WA, USA) was resuspended vigorously and 100 μl injected subcutaneously into the tail base. Animals were randomized into six groups (0.5% methylcellulose as vehicle, *n* = 7; CKD-506, 3 mg/kg, *n* = 8; CKD-506, 10 mg/kg, *n* = 8; CKD-506, 30 mg/kg, *n* = 8; CKD-506, 50 mg/kg, *n* = 8; and CKD-506, 100 mg/kg, *n* = 8). Each group received vehicle or oral CKD-506 once a day from day − 1 to day 16 relative to the injection. The severity of arthritis was assessed on days 9, 13, and 16 after injection of complete Freund’s adjuvant. Thereafter, rats were sacrificed.

### Arthritis assessment

The severity of arthritis was evaluated by scoring each joint (digits, metatarsal bones, and tarsal bones) as follows: 0, no swelling or erythema; 1, slight swelling and/or erythema; 2, low to moderate edema; 3, pronounced edema with limited joint usage; and 4, excess edema with joint rigidity. The clinical scores of four joints were summed to generate a total score for each animal.

### Statistical analysis

Data are presented as the mean ± SEM. Group in vitro experiments were compared using *t* tests. Clinical scores of treatment groups over time were compared using repeated measure analysis of variance (RM-ANOVA). All statistical analyses were performed in Prism software (GraphPad, La Jolla, CA, USA). *p* values < 0.05 were considered statistically significant.

## Results

### HDAC6 mediates pro-inflammatory responses via NF-κB and AP-1 signaling

**Fig. 1:**
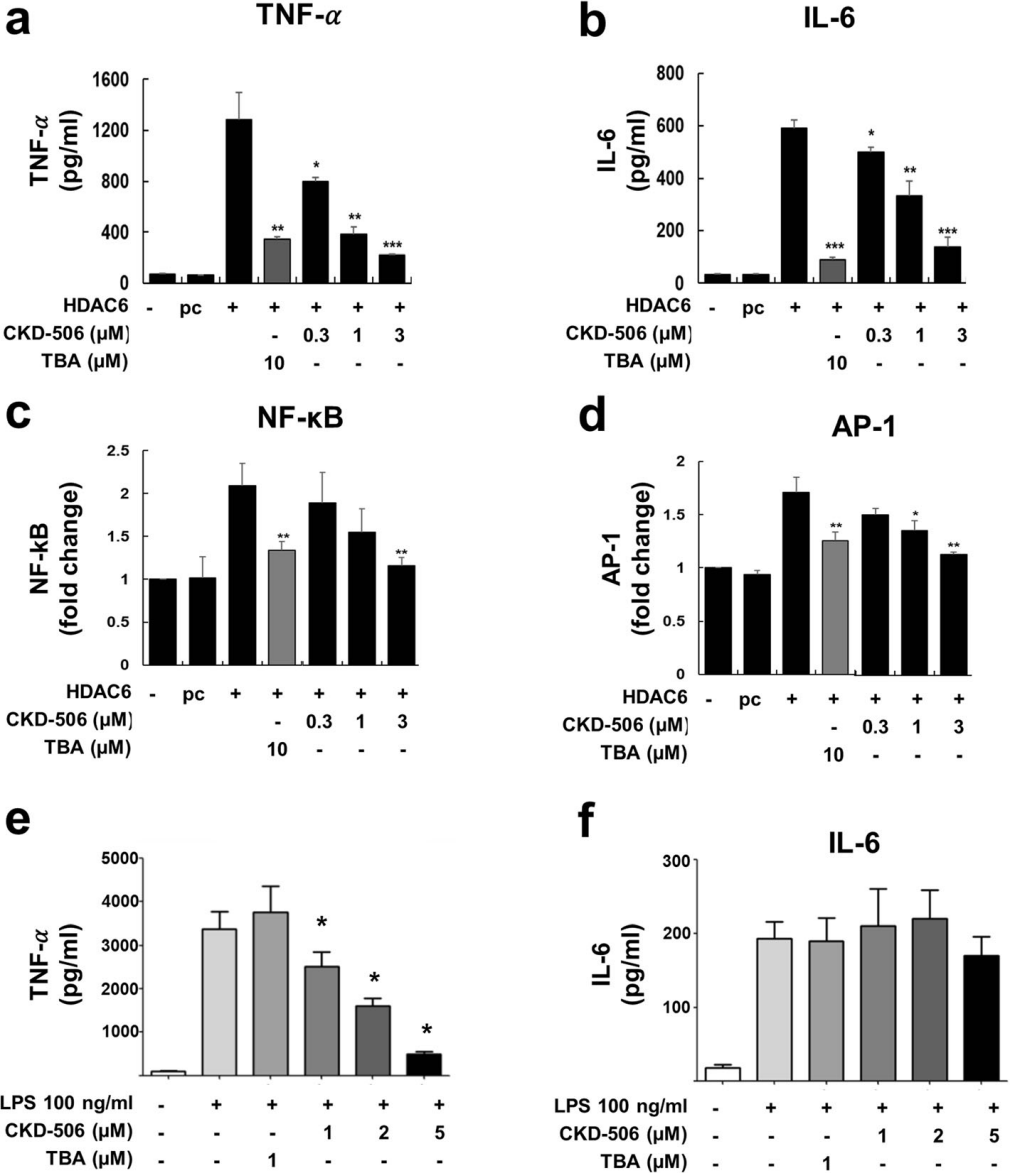
HDAC6 inhibitors suppress HDAC6-induced inflammatory responses. **a**, **b** Primary peritoneal macrophages (*n* = 3) were pretreated for 1 h with the indicated concentrations with CKD-506 or tubastatin (TBA) and then transfected with a control (pcDNA3.1 [pc]) or HDAC6 expression vector (1 μg/ml). At 48 h post-transfection, the levels of TNF-α (**a**) and IL-6 (**b**) in the culture medium were measured by ELISA. **c**, **d** RAW 264.7 cells (*n* = 3) were pretreated for 1 h with CKD-506 and then transiently co-transfected with an NF-κB (**c**) or AP-1 (**d**) promoter-luciferase expression vector, a β-galactosidase plasmid (pCMV-lacZ), and a control or HDAC6 expression vector. After 48 h, the luciferase activity in transfected cells was determined. Luciferase activity was normalized to that of β-galactosidase and expressed as -fold change over the control level. Data are expressed as the mean ± SEM of three independent experiments. *p < 0.05, **p < 0.01, and ***p < 0.001, compared with HDAC6-transfected cells. **e**, **f** PBMCs from RA patients (*n* = 5) were pretreated for 1 h with increasing concentrations of CKD-506 and then stimulated with LPS (100 ng/ml). Production TNF-α and IL-6 in the supernatant was measured using an ELISA. All data represent the mean value ± SEM. *p < 0.05, **p < 0.01 compared with no CKD-506 treatment. pc, plasmid control; TNF, tumor necrosis factor; IL, interleukin; TBA, tubastatin

First, we examined the effects of CKD-506 on production of pro-inflammatory cytokines and related signaling pathways in peritoneal macrophages transfected with an HDAC6 expression vector. Overexpression of HDAC6 increased spontaneous production of TNF-α and IL-6. By contrast, CKD-506 decreased production of TNF-α and IL-6 in a dose-dependent manner (Fig. 1a, b). Next, we analyzed the effect of CKD-506 on NF-κB and AP-1 promoter activity in HDAC6-transfected RAW 264.7 cells. Pretreatment of HDAC6-transfected cells with CKD-506 reduced NF-κB and AP-1 promoter activity in a dose-dependent manner (Fig. 1c, d). CKD-506 suppressed production of TNF-α, but not that of IL-6, by PBMCs from RA patients in response to LPS stimulation (Fig. 1e, f). These results suggest that CKD-506 inhibits HDCA6-mediated production of pro-inflammatory cytokines by regulating NF-κB and AP-1 signaling cascades. Of note, tubastatin A, another HDAC6 inhibitor, showed a similar effect.

### CKD-506 suppresses metalloproteinase and cytokine/chemokine production by FLS

**Fig. 2:**
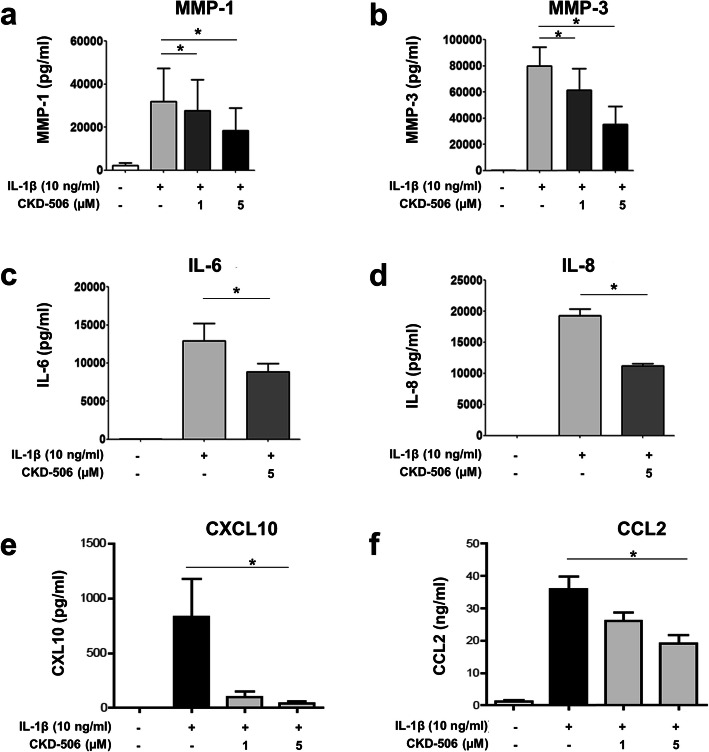
CKD-506 suppresses production of tissue-degradative enzymes, inflammatory cytokines, and chemokines by fibroblast-like synoviocytes (FLS). RA FLS (*n* = 3) were pretreated for 1 h with CKD-506 and then activated for 24 h with IL-1β (10 ng/ml). Then, the amounts of MMP-1 (**a**), MMP-3 (**b**), IL-6 (**c**), IL-8 (**d**), CXCL10 (**e**), and CCL2 (**f**) in the supernatant were measured in an ELISA. Data represent the mean value ± SEM. *p < 0.05

RA FLS were pretreated with increasing concentrations of CKD-506 and then stimulated with IL-1β. In response to IL-1β, FLS produced a large amount of metalloproteinases (MMP-1 and MMP-6), IL-6, and IL-8, along with chemokines CXCL10 and CCL2. Pretreatment with CKD-506 significantly reduced production of all of the above (Fig. 2).

### CKD-506 improves Treg function

**Fig. 3:**
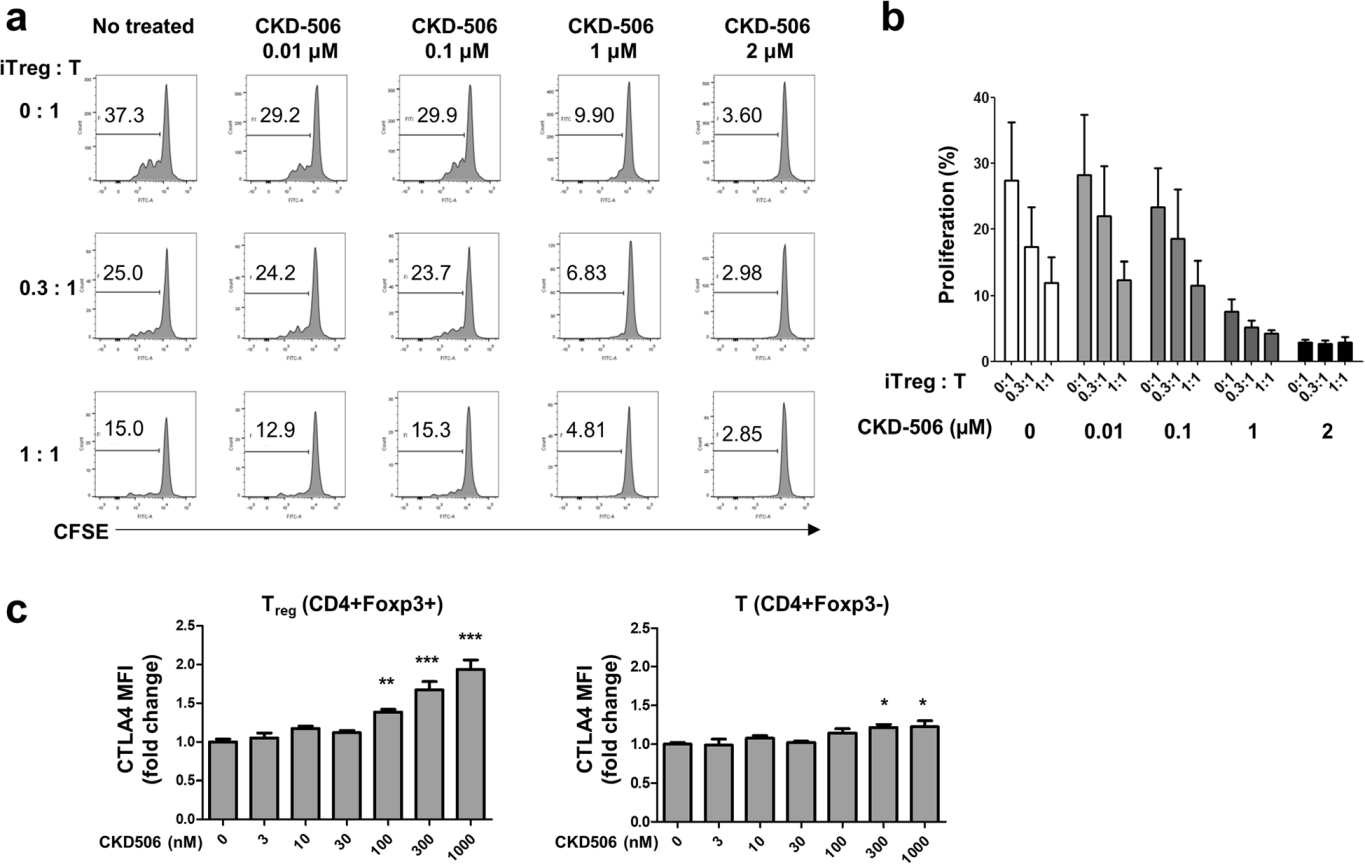
CKD-506 augments Treg-mediated suppression of T cell proliferation. Induced Treg cells (iTreg) from RA patients (*n* = 3) were co-cultured for 72 h at different ratios with CFSE-labeled T cells from healthy controls in the presence of an HDAC6 inhibitor and CD3/28 Dynabeads, and the proliferation of T cells was examined by FACS. **a** Representative FACS data from three independent experiments are shown. **b** T cell proliferation under different ratio of iTreg to T cells. **c** Changes in expression of CTLA4 by iTreg and T cells after treatment with CKD-506. *p < 0.05, **p < 0.01, ***p < 0.001 vs. no treatment

We investigated whether CKD-506 improves impaired Treg function in RA patients. First, iTregs from RA patients were co-cultured with CFSE-labeled T cells from healthy donors in the presence of increasing concentrations of CKD-506. In the absence of CKD-506, T cell proliferation decreased as the ratio of iTreg to T cells increased. CKD-506 potentiated inhibition of T cell proliferation. Interestingly, CKD-506 inhibited T cell proliferation even in the absence of iTregs. CKD-506 increased expression of CTLA4 by Foxp3+ iTregs and Foxp3− T cells (Fig. 3).

### CKD-506 prevents experimental arthritis in a murine model

**Fig. 4:**
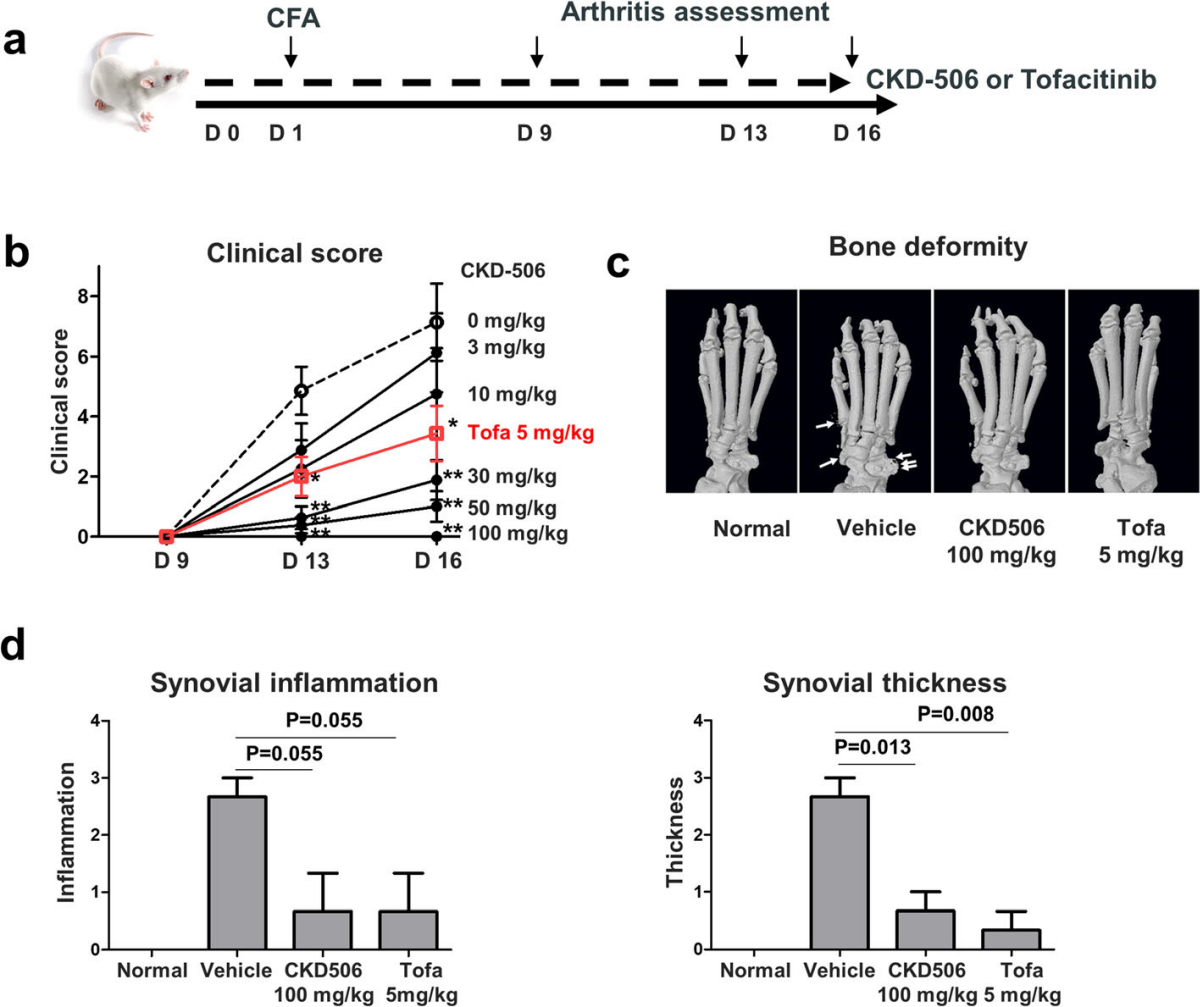
CKD-506 prevents experimental arthritis in an AIA rat model. **a** Experimental design. Rats with experimentally induced arthritis were treated with daily oral CKD-506. Severity of arthritis was assessed on days 9, 13, and 16 after CFA injection. Rats were sacrificed thereafter. **b** Clinical scores over the treatment period are shown according to CKD-506 treatment dose. Groups were compared using repeated measure ANOVA, followed by post hoc analysis with Bonferroni corrections. **c** Representative 3D reconstructed image of the hind foot. White arrows indicate bone erosions. **d** The efficacy of CKD-506 at 100 mg/kg QD vs. tofacitinib 5 mg/kg (with respect to the effects on synovial inflammation and thickness) was compared. Data represent the mean value ± SEM. *p < 0.05, **p < 0.01 vs. CKD-506 at 0 mg/kg. CFA, complete Freund’s adjuvant; Tofa, tofacitinib

The efficacy of CKD-506 on inflammatory arthritis was evaluated in a murine arthritis model. Rats were treated with daily oral CKD-506 at 3, 10, 30, 50, and 100 mg/kg, or with tofacitinib at 5 mg/kg, from 1 day before to 16 days after CFA injection. The clinical arthritis scores started to rise on day 9 and continued to rise until day 16 (Fig. 4a). CKD-506 reduced the arthritis score on days 13 and 16 in a dose-dependent manner (Fig. 4b). CDK-506 at 100 mg/kg/day, and tofacitinib at 5.0 mg kg/day, had comparable effects with respect to inhibiting synovial inflammation and bone destruction (Fig. 4c, d). Of note, serum levels of anti-CCP antibody on day 16 tended to be lower in rats treated with CKD-506 (Supplementary figure S1).

### CKD-506 ameliorates experimental arthritis and has synergistic effect of CDK-506 with methotrexate in a murine model

**Fig. 5:**
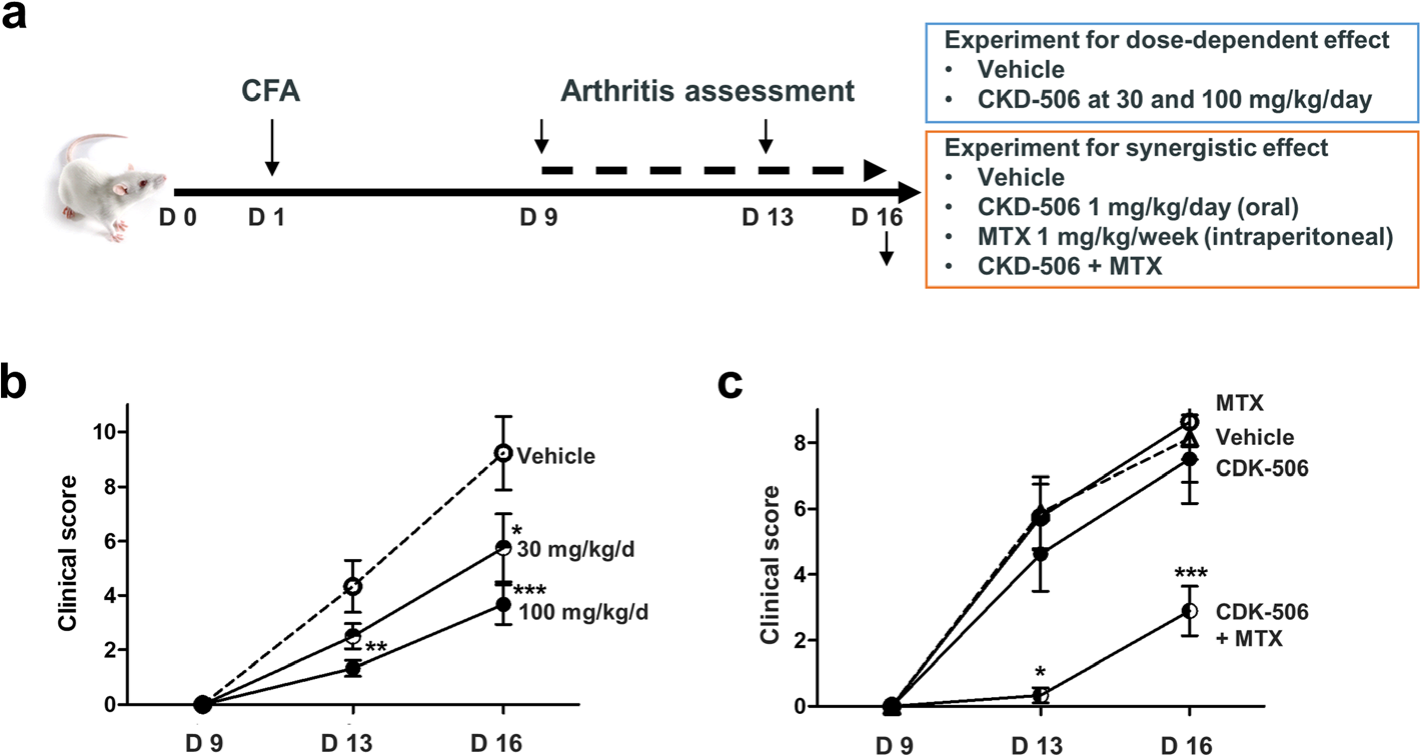
CKD-506 ameliorates experimental arthritis and shows a synergistic effect with MTX in an AIA rat model. **a** After induction of arthritis, rats were treated with CKD-506 with or without MTX starting 9 days after CFA injection. **b** Anti-arthritic effects by CKD-506 at 0 (vehicle treatment), 30, and 100 mg/kg/day were depicted. **c** Anti-arthritic effects of low dose MTX 1 mg/kg/week, low dose CKD 1 mg/kg/day, and their combination treatment were shown. Data represent the mean value ± SEM. *p < 0.05, **p < 0.01, ***p < 0.001 vs. vehicle treatment. Groups were compared using repeated measure ANOVA, followed by post hoc analysis with Bonferroni corrections

To test the therapeutic effect of CKD-506, rats were treated with daily CKD-506 at 0, 30, and 100 mg/kg/day from 9 to 16 days after CFA injection (Fig. 5a). CKD-506 ameliorated the arthritis score in a dose-dependent manner (Fig. 5b). AIA rats were treated with a sub-therapeutic dose of CKD-506 (1 mg/kg/day) or methotrexate (MTX; 1 m/kg/week), or both. CKD-506 or MTX alone did not improve the clinical score compared with vehicle treatment. However, the combination of CKD-506 and MTX reduced the arthritis score significantly on days 13 (5.8 + 3.1 vs. 0.3 + 0.2 *p* < 0.001) and 16 (8.2 + 3.7 vs. 2.9 + 2.3, *p* = 0.003), respectively (Fig. 5c).

## Discussion

A hallmark of RA is chronic inflammation and destruction of joints. Dysfunction at various steps of the complex network comprising humoral and cellular immune responses results in perpetuation of inflammatory activation during development of RA, in which pro-inflammatory autoimmune responses are not adequately regulated by anti-inflammatory mechanisms [3]. Here, we show that CKD-506, a novel specific HDAC6 inhibitor, suppressed inflammatory responses by macrophages, improved Treg function, and inhibited inflammatory responses and the joint-destructive properties of FLS in vitro. In vivo, CKD-506 attenuated arthritis in a murine RA model with efficacy comparable with that of tofacitinib. In addition, CKD-506 had a synergistic effect when combined with methotrexate (both drugs were used at a dose that was ineffective when each was used alone).

Pan-HDAC inhibitors exhibit strong anti-inflammatory and anti-proliferative properties in several immune-mediated and hematologic malignancies; however, they have a narrow therapeutic margin due to side effects such as memory loss, cytopenia, and diarrhea [7, 14, 22–24]. In addition, epigenetic changes might have an unpredictable, long-lasting impact on the immune and non-immune cells even after HDAC inhibition is stopped. HDAC6 is localized predominantly to the cytoplasm where it deacetylates cytoplasmic non-histone proteins such as HSP90 and α-tubulin [15, 25, 26]. Hyperacetylation of α-tubulin has anti-inflammatory effects [23, 24]. CKD-506 is a highly specific inhibitor of HDAC6; as such, it has pleiotropic effects on both immune cells and FLS, which constitute the main cellular infiltrates in inflamed RA joints [20]. Here, we found that CKD-506 decreased secretion of pro-inflammatory cytokines TNF-α and IL-6 by macrophages in part via suppression of the NF-κB and AP-1 pathways (Fig. 1). Overexpression of HDAC6 during an inflammatory response upregulated NADPH oxidase with increased generation of cytosolic reactive oxygen species (ROS). The generated ROS promotes activation of the NF-κB and AP-1 signaling pathways with subsequent transcription and production of inflammatory cytokines [17]. Intriguingly, CKD-506 did not significantly suppress IL-6 production by the LPS-stimulated RA PBMCs (Fig. 1f). This is likely due to the relatively low IL-6 production by PBMCs which results in “less suppressive effect” of CKD-506.

CKD-506 suppressed immune responses directly by suppressing proliferation of activated T cells and indirectly by augmenting the function of regulatory T cells (Fig. 3). Restoration of defective Treg function is crucial for maintenance of self-tolerance and for preventing aggressive autoimmune cells from destroying tissues in RA joints [27]. Transcription factor Forkhead box P3 (Foxp3) is crucial for the development and function of Tregs. In Treg cells, acetylation of Foxp3 is an important post-translational mechanism to regulate its concentration since acetylated Foxp3 is protected from proteosomal degradation. HDAC6 inhibitor therefore increases Foxp3 half-life and so enhances suppressive function of Treg cells [28, 29]. It is possible that T cells become more sensitive to the Treg-mediated inhibition by CKD-506. Therefore, CKD-506 suppresses T cell proliferation by multiple mechanisms.

The inflammatory environment of RA transforms normal resident synoviocytes into tissue-destructive FLS, which produce large amounts of IL-6, IL-8, and extracellular matrix proteinases MMP-1 and MMP-3 [30, 31]. CKD-506 markedly inhibits joint-destructive FLS [18, 29]. The multicellular effects of HDAC6 inhibition by CKD-506 might contribute to the anti-arthritic effects observed in the murine RA model; these effects were comparable with those of tofacitinib, a JAK/STAT signaling inhibitor with proven clinical efficacy (Fig. 4). Of note, CKD-506 exhibited both prophylactic and therapeutic effects; CKD-506 treatment before and after induction of arthritis showed anti-arthritic effects although the prophylactic treatment ameliorated the arthritis score better than therapeutic treatment. Strikingly, the combination of low (sub-therapeutic) dose CKD-506 and low (sub-therapeutic) dose methotrexate had a synergistic therapeutic effect on the arthritis severity in the murine model (Fig. 5). A previous report shows that methotrexate restores Treg function by demethylating the upstream enhancer of FoxP3, which might also potentiate restoration of impaired Treg cell function by CKD-506 [32]. While the mechanism underlying synergism needs further investigation, combination treatment might enable reduction of the dose of CKD-506 and reduce the risk of side effects. Long-term drug safety is of particular interest since patients with RA require life-long treatment [33]. Taken together, inhibiting multiple cell types with the novel HDAC6 inhibitor CKD-506 is promising, and the results are consistent with the observed anti-arthritic effects of other HDAC6 inhibitors [34, 35]. This supports also the anti-inflammatory effect of CKD-506 as a novel therapeutic agent to treat diverse autoimmune diseases such as SLE and inflammatory bowel disease [20, 21].

A main limitation of the study is that we did not investigate the exact mechanism by which the HDAC6 inhibitor affects multiple steps in the inflammatory response; indeed, many cytosolic and non-cytosolic proteins are substrates for HDAC6, so examining the mechanism is important.

## Conclusions

In conclusion, CKD-506, a novel HDAC6 inhibitor, regulates innate and adaptive immune responses and ameliorates experimental arthritis. CKD-506 has potential as a novel treatment option for treatment of RA.

## Supplementary information

**Additional file 1 : Supplementary figure S1.** Serum anti-CCP antibody titers were decreased by CKD-506. Serum anti-CCP levels were measured on Day 16 after CFA injection. Data represent the mean value ± SEM. *p < 0.05, **p < 0.01, ***p < 0.011 vs. CKD-506 0 mg/kg.)

## Data Availability

Not applicable.

## Abbreviations

AIA: Adjuvant-induced arthritis
CFSE: Carboxyfluorescein diacetate succinimidyl ester
ELISA: Enzyme-linked immunosorbent assay
FLS: Fibroblast-like synoviocytes
HDAC: Histone deacetylase
IL: Interleukin
MMP: Matrix metalloproteinase
MTX: Methotrexate
PBMC: Peripheral blood mononuclear cell
iTreg: Induced regulatory T cell
RA: Rheumatoid arthritis
TNF: Tumor necrosis factor
