# Supplementary material for: Therapeutic potential of CKD-506, a novel selective histone deacetylase 6 inhibitor, in a murine model of rheumatoid arthritis
Source: Arthritis Res Ther. 2020 Jul 25;22:176. doi: 10.1186/s13075-020-02258-0 (PMC7382061; doi:10.1186/s13075-020-02258-0)
Supplement: Supplementary file 1 — Additional file 1 : Supplementary figure S1. Serum anti-CCP antibody titers were decreased by CKD-506. Serum anti-CCP levels were measured on Day 16 after CFA injection. Data represent the mean value ± SEM. *p < 0.05, **p < 0.01, ***p < 0.011 vs. CKD-506 0 mg/kg.) [file 13075_2020_2258_MOESM1_ESM.docx]

**Supplementary Material**

**Supplementary figure S1. Serum anti-CCP antibody titers were decreased by CKD-506.** Serum anti-CCP levels were measured on Day 16 after CFA injection. Data represent the mean value ± SEM. *p < 0.05, **p <0.01, ***p <0.011 *vs.* CKD-506 0 mg/kg.

**
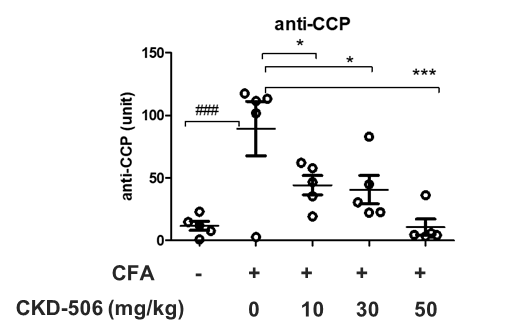
**
